# Supplementary material for: Three dimensional band-filling control of complex oxides triggered by interfacial electron transfer
Source: Nat Commun. 2021 Apr 27;12:2447. doi: 10.1038/s41467-021-22790-0 (PMC8079372; doi:10.1038/s41467-021-22790-0)
Supplement: Supplementary file 1 — Supplementary Information [file 41467_2021_22790_MOESM1_ESM.pdf]

# Supplementary Information:

## Three dimensional band-filling control of complex oxides triggered by interfacial electron transfer


Meng Meng[1], Yuanwei Sun[2], Yuehui Li[2], Qichang An[1,3], Zhenzhen Wang[1,3], Zijian Lin[1,3], Fang Yang[1], Xuetao Zhu[1,3,4], Peng Gao[3,5*], and Jiandong Guo[1,3,4,6*]

[1] *Beijing National Laboratory for Condensed Matter Physics and Institute of Physics, Chinese Academy of Sciences, Beijing 100190, China*

[2] *International Center for Quantum Materials, and Electron Microscopy Laboratory, School of Physics, Peking University, Beijing 100871, China*

[3] *School of Physical Sciences, University of Chinese Academy of Sciences, Beijing 100049, China*

[4] *Songshan Lake Materials Laboratory, Dongguan, Guangdong 523808, China*

[5] *Collaborative Innovation Center of Quantum Matter, Beijing 100871, China*

[6] *Beijing Academy of Quantum Information Sciences, Beijing 100193, China*

---

[*]To whom correspondence should be addressed.

Jiandong Guo: jdguo@iphy.ac.cn and Peng Gao: p-gao@pku.edu.cn.

**Supplementary Note 1: Thin film growth and characterization**

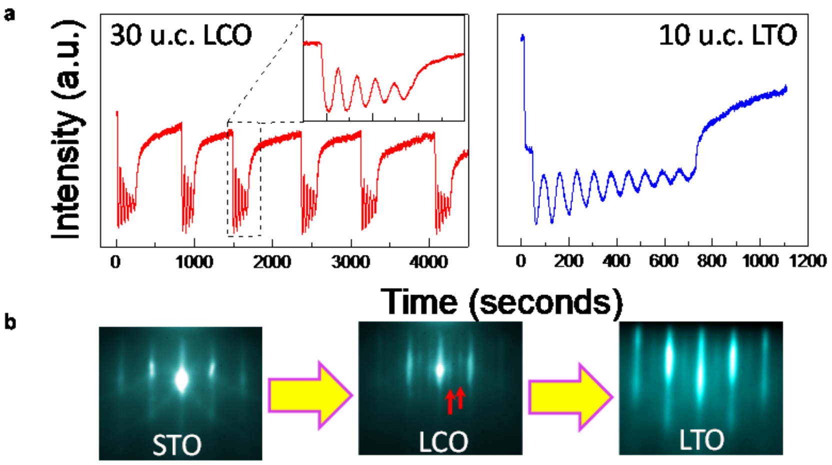


**Supplementary Figure 1. a**. Evolution of the RHEED intensity during the growth of 30 u.c. LCO and 10 u.c. LTO. To maintain the high-quality of the film, we used the interrupted deposition method to grow LCO. The laser pulse was interrupted and the sample was annealed after each growth of 5 u.c. LCO (Inset of **a,** left panel). **b**. In-situ RHEED patterns of STO substrate, as-grown LCO, and as-grown LTO. The red arrows indicate surface reconstruction of LCO, confirming the flatness and good crystallinity.

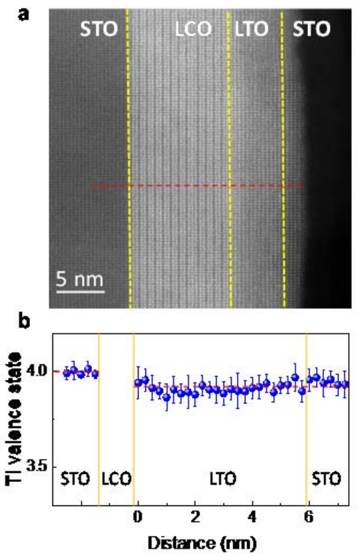


**Supplementary Figure 2. a.** The HAADF-STEM image of C30/T15 capped with STO. **b.** The calculated Ti oxidation states of C30/T15 as a function of position determined by Ti *L* edge peak positions in the spatially resolved STEM-EELS spectra collected along the red dashed line shown in **a**. The position of the LCO/LTO interface is set to be zero. The error bars are determined from the errors introduced in background subtraction, data fluctuation and standard deviation of spectra fitting.

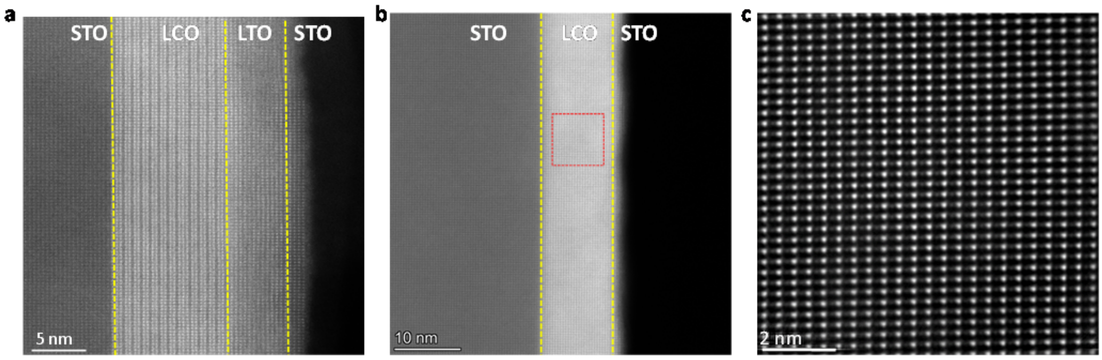


**Supplementary Figure 3.** HAADF-STEM images of **a** C30/T15 and **b** C30/T0 taken along the STO [110] direction. **c** The zoom-in image (red dashed square in **b**) of the LCO layer in the C30/T0.

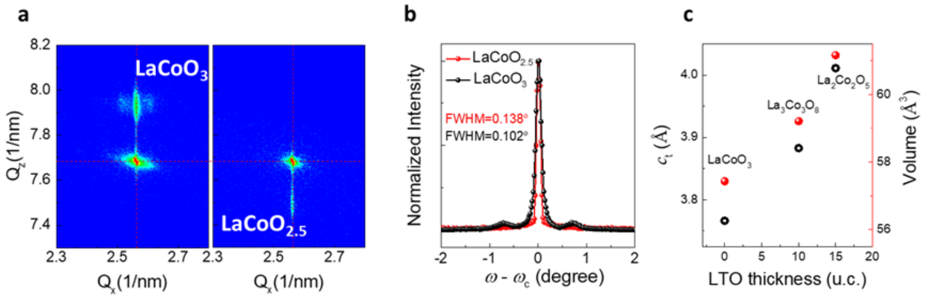


**Supplementary Figure 4. a**. Reciprocal space map (103) of the C30/T0 and C30/T15 heterostructures on STO substrate. **b**. Rocking curve scans of the C30/T0 and C30/T15 heterostructures, indicating that the BM $La_2Co_2O_5$ has even better crystallinity than the perovskite LCO. **c**. The out-of-plane lattice constant and the volume of the pseudotetragonal unit cell of the C30/T*n* samples as a function of the LTO thickness.

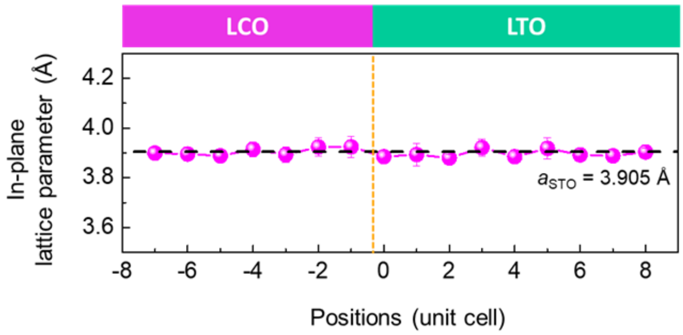


**Supplementary Figure 5.** In-plane lattice parameters of C30/T15 as a function of distance from the LCO/LTO interface indicating by the dashed orange line. The dashed black line indicates the in-plane lattice constant of bulk STO (3.905 Å). The error bar shows the standard deviations of the averaged measurements.

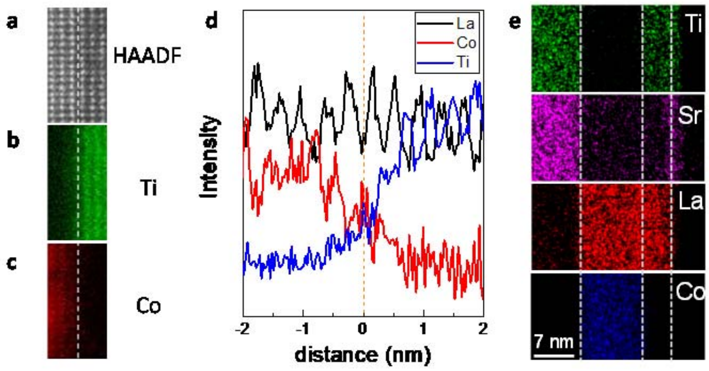


**Supplementary Figure 6.** STEM/EELS elemental maps of C30/T15 near the LCO/LTO interface. **a.** HAADF-STEM image taken along STO [110] direction. EELS elemental maps from **b** Ti and **c** Co. The white dashed line marks the LCO/LTO interface. **d.** The signal intensity of Ti and Co as a function of distance. The LCO/LTO interface is very sharp with small amounts of Ti/Co intermixing that is around 1 u.c. near the interface. Together with the high resolution HAADF image shown in Supplementary Figure 3c, we could exclude the formation of $La_2CoTiO_6$ double perovskite phase near the LCO/LTO interface. **e.** Large area STEM/EDX maps of the C30/T15.

**Supplementary Note 2: First principle calculations**

We used first principle calculations with density functional theory by the *Vienna ab initio* simulation package code [1] to evaluate the energy lowering as the perovskite $LaCoO_3$ changing into the OVO phase and releasing $O^{2-}$ ions upon electrons transferring from the $LaTiO_3$. For a simple illustration, the total energies of two structures with the identical atoms and charges were compared: $2(LaCoO_3+e)$ and $La_2Co_2O_5+O^{2-}$. The lattice parameters were taken as the relaxed values from the *Material Project* (https://materialsproject.org) and were fixed in the current calculations: $a = b = 5.541$ Å, $c = 13.260$ Å for the perovskite $LaCoO_3$ and $a = 5.458$ Å, $b = 15.792$ Å, $c = 5.846$ Å for the brownmillerite $La_2Co_2O_5$. A cutoff energy of 500 eV and an automatic gamma *k*-point mesh of 5 × 5 × 5 were used to ensure that the energy converged within 10 meV/cell for $LaCoO_3$ and 100 meV/cell for $La_2Co_2O_5$. The PAW-LDA pseudopotential was employed and the *U* values were chosen as 3.8 eV and 8 eV on the *3d* orbit of Co and La, respectively. The energy of an isolated $O^{2-}$ ion was calculated in a 12 Å × 13 Å × 14 Å vacuum slab. A cutoff energy of 450 eV and 1 × 1 × 1 Monkhorst Pack *k*-point mesh were applied. The energy convergence reached 1 meV/cell.

**Supplementary Table 1.** The calculation results of total energy.

| Structure | $E_{total}$ (eV) |
|---|---|
| $\mathbf{La_2Co_2O_6}$ | -76.08 |
| $\mathbf{(La_2Co_2O_6) + 2e^-}$ | -68.53 |
| $\mathbf{La_2Co_2O_5 + O^{2-}}$ | -70.37 |

**Supplementary Note 3: Control samples**

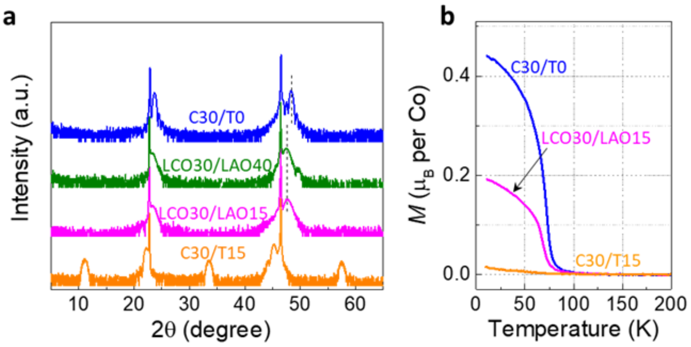


**Supplementary Figure 7. a**. X-ray diffraction data of C30/T0, LCO(30 u.c.)/oxygen-deficient LAO(15 u.c.), LCO(30 u.c.)/oxygen-deficient LAO(40 u.c.), and C30/T15. **b**. Temperature dependent magnetization of C30/T0, LCO(30 u.c.)/oxygen-deficient LAO(15 u.c.), and C30/T15. For the LCO(30 u.c.)/ oxygen-deficient LAO(15 and 40 u.c.) reference sample, LAO was fabricated using the same growth conditions for LTO, resulting to an oxygen-deficient LAO. Reference samples with the oxygen-deficient LAO capped have a perovskite LCO with a larger out-of-plane lattice parameter compared with that of C30/T0. The MT data reveals a reduced magnetization due to oxygen-vacancy. So capping oxygen-deficient LAO only results to the increase of oxygen vacancy content in the buried LCO. However, such a peak shift does not change by increasing the LAO thickness to 40 u. c.. It can be concluded that the oxygen scavenging induced by the capping layer cannot be the key reason for the results observed in the current work.

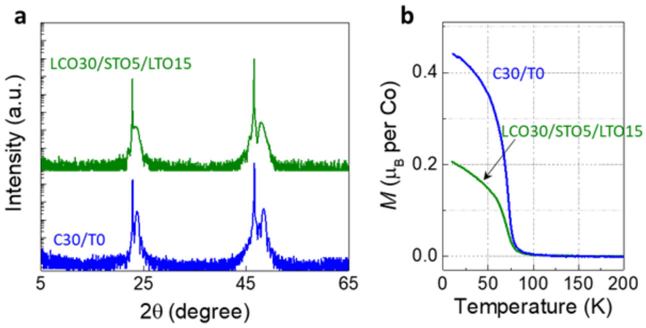


**Supplementary Figure 8. a**. X-ray diffraction data of C30/T0 and LCO(30 u.c.)/STO(5 u.c.)/LTO(15 u.c.). **b**. Temperature dependent magnetization of C30/T0 and LCO(30 u.c.)/STO(5 u.c.)/LTO(15 u.c.). For the LCO(30 u.c.)/STO(5 u.c.)/LTO(15 u.c.) reference sample, the STO blocking layer was grown using the same conditions for LTO. This sample shows perovskite structure with some oxygen vacancies as in the LCO(30 u.c.)/oxygen-deficient LAO(15 and 40 u.c.).

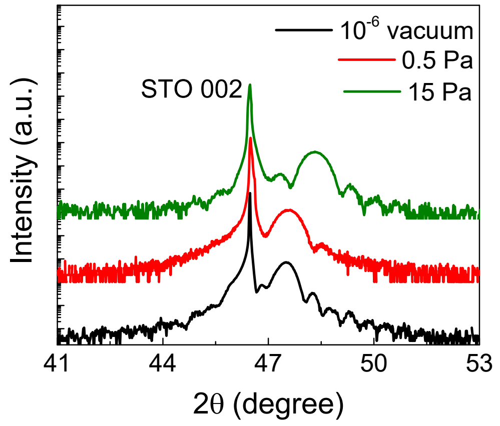


**Supplementary Figure 9.** X-ray diffraction data of LCO (30 u.c.)/STO (10 u.c.) grown under different oxygen partial pressures.

We have grown LCO(30 u.c.)/STO(10 u.c.) on STO (001) substrates under various oxygen partial pressures. As shown in the Supplementary Figure 9, even grown under the pressure as low as $1\times10^{-6}$ Pa (vacuum), LCO still maintains the perovskite structures. Only STO as an oxygen sponge could not lead to the observed phenomena. This results can also exclude the possibility that ordered vacancy phases form spontaneously in LCO at high-temperature low-pressure conditions. This observation fits in the scenario that growth of LCO thin films even under extreme oxygen deficient environment would yield the most thermodynamically stable phase.

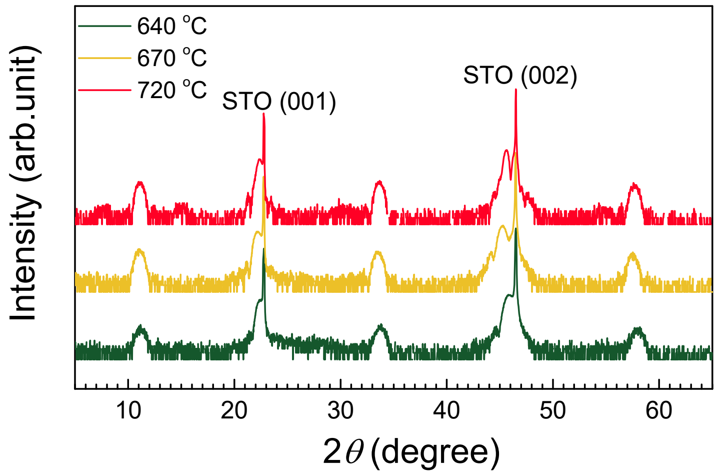


**Supplementary Figure 10.** X-ray diffraction data of C30/T15 heterostructures using different growth temperature during LTO layer deposition.

We have grown C30/T15 heterostructures at different temperatures during the LTO layer deposition (640 to 720 °C). Supplementary Figure 10 shows the corresponding XRD results. All the three C30/T15 samples are in the brownmillerite $La_2Co_2O_5$ phase. We note that LTO has a narrow growth window — we cannot obtain good crystallinity of LTO out of the above temperature range manifested by the rapid decay of the RHEED oscillation and RHEED patterns during the growth. Within the range, we did not observe any change of the ordering of the octahedra/tetrahedra arrangement.

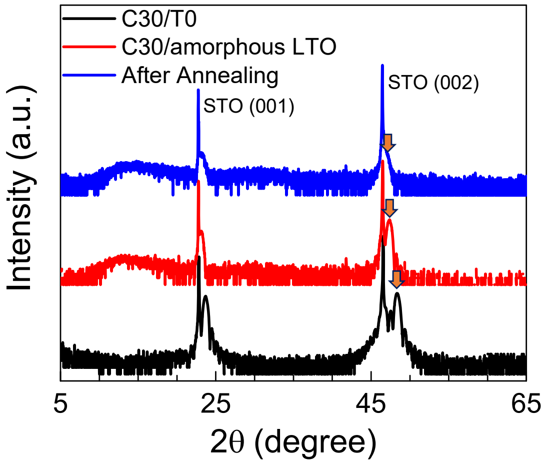


**Supplementary Figure 11.** X-ray diffraction data of LCO(30 u.c.), as-grown and annealed LCO(30 u.c.)/amorphous LTO.

We have grown amorphous LTO on high-quality LCO (30 u.c.) at ambient temperature and under high vacuum ($1\times10^{-6}$ Pa). For crystalline LTO grown at 670 °C, as monitored by RHEED intensity oscillations, the growing rate was around 140 pulses per u.c.. While for the current reference sample, the repetition rate was 2 Hz and the total pulse was ~ 4200, leading to around 12-nm-thick amorphous LTO. Supplementary Figure 11 shows the XRD result of LCO(30 u.c.)/ amorphous LTO. The LCO (002) from LCO(30 u.c.)/amorphous LTO shifts to lower angle compared with that from LCO (30 u.c.), indicating the enlarged out-of-plane lattice parameter

and the creation of oxygen vacancies because of the redox reaction by amorphous LTO capping. However, we did not observe the topotactic phase transition of LCO in this reference sample, as observed in the C30/T10 and C30/T15 heterostructures. Therefore, we could exclude the reduction of LCO is only due to the redox reaction or oxygen absorber of the LTO film [2]. Moreover, the LCO/amorphous LTO sample has been annealed at 670 °C for one hour. LCO still maintains the perovskite structure after the annealing, further excluding the redox reaction of LCO due to LTO.

**Supplementary References:**

[1] Kresse, G. & Furthmüller, J. Phys. Rev. B 54, 11169 (1996).
[2] Y. Chen et al. Nano Lett., 11, 3774, (2011).
